# Outcome measures for hand function naturally reveal three latent domains in older adults: strength, coordinated upper extremity function, and sensorimotor processing

**DOI:** 10.3389/fnagi.2015.00108

**Published:** 2015-06-05

**Authors:** Emily L. Lawrence, Sudarshan Dayanidhi, Isabella Fassola, Philip Requejo, Caroline Leclercq, Carolee J. Winstein, Francisco J. Valero-Cuevas

**Affiliations:** ^1^Brain-Body Dynamics Laboratory, Department of Biomedical Engineering, University of Southern CaliforniaLos Angeles, CA, USA; ^2^Brain-Body Dynamics Laboratory, Division of Biokinesiology and Physical Therapy, University of Southern CaliforniaLos Angeles, CA, USA; ^3^Institut de la Main, Clinic JouvenetParis, France; ^4^Rancho Los Amigos National Rehabilitation CenterDowney, CA, USA

**Keywords:** aging, sensorimotor processing, dexterity, hand, strength

## Abstract

Understanding the mapping between individual outcome measures and the latent functional domains of interest is critical to a quantitative evaluation and rehabilitation of hand function. We examined whether and how the associations among six hand-specific outcome measures reveal latent functional domains in elderly individuals. We asked 66 healthy older adult participants (38F, 28M, 66.1 ± 11.6 years, range: 45–88 years) and 33 older adults (65.8 ± 9.7 years, 44–81 years, 51 hands) diagnosed with osteoarthritis (OA) of the carpometacarpal (CMC) joint, to complete six functional assessments: hand strength (Grip, Key and Precision Pinch), Box and Block, Nine Hole Pegboard, and Strength-Dexterity tests. The first three principal components suffice to explain 86% of variance among the six outcome measures in healthy older adults, and 84% of variance in older adults with CMC OA. The composition of these dominant associations revealed three distinct latent functional domains: strength, coordinated upper extremity function, and sensorimotor processing. Furthermore, in participants with thumb CMC OA we found a blurring of the associations between the latent functional domains of strength and coordinated upper extremity function. This motivates future work to understand how the physiological effects of thumb CMC OA lead upper extremity coordination to become strongly associated with strength, while dynamic sensorimotor ability remains an independent functional domain. Thus, when assessing the level of hand function in our growing older adult populations, it is particularly important to acknowledge its multidimensional nature—and explicitly consider how each outcome measure maps to these three latent and fundamental domains of function. Moreover, this ability to distinguish among latent functional domains may facilitate the design of treatment modalities to target the rehabilitation of each of them.

## Introduction

The hand is vital for human activities and independent living and influences the quality of task performance, especially those requiring dexterity (Light et al., 1999). As such, quantifying hand function is central to research and clinical care and numerous outcome measures have been developed to evaluate treatment effectiveness and ultimately improve medical care (Smith, 1961; Cromwell, 1976; Walker et al., 1978; Mathiowetz et al., 1985a; Hume et al., 1990; Marx et al., 1999; Light et al., 2002; Oxford Grice et al., 2003). The central question here is, What should we use to quantify hand function considering that that we have so many choices of assessment tools and even more outcome measures stemming from those tools? It stands to reason that the multi-dimensional nature of hand function would require multiple outcome measures for accurate assessment of ability. But the shear number of available outcome measures creates a false sense of high-dimensionality. This motivates us to evaluate the associations, commonalities, and dissociations among outcome measures, and their ability to reveal latent functional domains. We propose that understanding the mapping between individual outcome measures and the latent functional domains of interest is critical to the quantitative evaluation and rehabilitation of hand function. To clarify, we define latent functional domains as the hidden dimensions underlying hand function. We believe this approach will address and help resolve the debate over the merits of available outcome measures.

In the motor function community, some advocate the preeminence of measures of hand strength or joint range of motion (Light et al., 1999). Others prefer outcome measures geared towards Activities of Daily Living (ADLs) that feature coordinated upper extremity function (Light et al., 1999) such as time limited measures (i.e., amount completed in a given time) like the Box and Blocks test (BBT; Mathiowetz et al., 1985b) and the Crawford Small Parts Dexterity test (Boyle and Santelli, 1986). Yet still others emphasize work limits (i.e., time to completion) such as the Nine Hole Peg test (NHPT; Oxford Grice et al., 2003) and the Functional Dexterity test (Mathiowetz et al., 1985b; van Lankveld et al., 1996). While all of these outcome measures have shown utility, it is recognized that they offer limited information (Light et al., 1999, 2002; Duff et al., 2015). As a result, new assessment tools were developed that include a battery of measures designed to assess a set of motor functional abilities like the Jebsen Taylor Hand Function (Jebsen et al., 1969) and TEMPA tests (Desrosiers et al., 1995). There are other measures focusing on sensory acuity like the Weber two-point discrimination (Dellon et al., 1987) and the AsTex sensitivity tests (Miller et al., 2009)—but sensorimotor control is difficult to test while disambiguating it from strength, coordinated upper extremity function, tactile and visual acuity, and speed. We stress that sensorimotor processing is integrative by definition, and must be considered independently of isolated motor or sensory function. One example of sensorimotor fingertip function is the ability to dynamically control the magnitudes and directions of force vectors, as quantified by the Strength-Dexterity (SD) test (Valero-Cuevas et al., 2003; Dayanidhi et al., 2013; Lawrence et al., 2014).

But the questions remain: what latent domains describe hand function and how do individual outcome measures relate to latent functional domains of interest? In fact, the International Classification of Functioning, Disability and Health (ICF) by the World Health Organization (2001) highlights the importance of quantifying latent functional domains related to body structure and function, activity, and participation, which clearly require several different assessment tools. Seen from this perspective it is difficult to define and justify a specific selection of—and hierarchy among—available assessment tools. Thus, several rehabilitation studies have begun to explore interactions among outcome measures (Hellström et al., 2003; Patterson et al., 2010; Hart and Bagiella, 2012; McDonough et al., 2013; Milot et al., 2013; Egan et al., 2014). Similarly, here we examine whether and how the interactions and associations among six commonly used outcomes measures reveal latent functional domains in: (i) healthy older adults; and (ii) older adults with thumb carpometacarpal osteoarthritis (CMC OA).

## Material and Methods

Sixty-six healthy adult participants (38F, 28M, 66.1 ± 11.6 years, range: 45–88 years) completed the following assessments that utilize varying levels of strength requirements with their dominant hand (described in detail below): BBT, NHPT, SD test, and measures of finger and hand strength (grip strength, key pinch, and precision pinch). We then asked 33 adult participants (65.8 ± 9.7 years, 44–81 years, 51 hands) diagnosed with and treated for CMC OA to complete the same assessments with their affected hand(s). These patients were evaluated at an average of 40 months after either surgical or conservative treatment by the same surgeon (author CL) at Institut de la Main, Clinique Jouvenet in Paris, France between September 2005 and December 2011. All participants gave their informed consent to the experimental protocols, which were approved by the Institutional Review Boards at Rancho Los Amigos National Rehabilitation Center and the University of Southern California. The assessments were performed during a single session and participants were allowed to rest as often as needed in between tests.

### Grip/Key Pinch/Precision Pinch Strengths

Hand and finger strength is often used as a measure of function in the upper extremity (Light et al., 1999). Grip, key, and precision (tip-to-tip) pinch strengths were measured using standard techniques (patient sitting with the upper arm by the side, elbow flexed to 90°, and forearm in neutral rotation) with calibrated grip and pinch meters (Jamar, Jackson, MO; Mathiowetz et al., 1985a). Participants completed three trials for each measure and the dependent variables were the highest value from the three trials.

### Box and Blocks Test

The BBT (Cromwell, 1976; Mathiowetz et al., 1985b) is a measure of coordinated upper extremity function (Trombly, 1983) that has been validated and used to assess numerous clinical conditions (Smith, 1961). Participants were asked to use one hand to move blocks, one at a time, from one compartment of a box to another that was separated by a divider. The dependent variable was the number of blocks transported in one minute.

### Nine-Hole Peg Test

The NHPT is a test of fine motor control featuring an emphasis on finger dexterity (Oxford Grice et al., 2003). For the NHPT, participants were asked to take narrow pegs from a shallow trough, one by one, and place them into the holes on the board, then remove the pegs from the holes, one by one and return them to the trough as quickly as possible (Oxford Grice et al., 2003). The time to complete the task, the dependent variable, was recorded with a stopwatch.

### Strength-Dexterity Test

The SD test is described in detail in prior publications (Valero-Cuevas et al., 2003; Dayanidhi et al., 2013; Dayanidhi and Valero-Cuevas, 2014; Lawrence et al., 2014). Briefly, it is a continuous measure that involves using the first digit and thumb to compress as far as possible a slender spring, prone to buckling. This requires control of fingertip motions and force vectors at very low force levels (2–3 N) and is informative of one’s level of neuromuscular control (Valero-Cuevas et al., 2003; Vollmer et al., 2010; Holmström et al., 2011; Dayanidhi and Valero-Cuevas, 2014; Lawrence et al., 2014). The spring is outfitted with miniature force sensors (Measurement Specialties, Hampton, VA) on either end to quantify the forces exerted by the fingertips. Participants were asked to compress the spring as much as possible and maintain steady state compression (hold phase) for 3–5 s for at least ten trials. Force data were sampled at 400 Hz with a data acquisition system (National Instruments, Austin, TX) and both recorded and processed with custom Matlab software (The Mathworks, Natick, MA). The three hold phases with the greatest mean compression forces were considered the dependent variables for analysis. Mean compression forces are indicative of ones ability to overcome instabilities during the hold phase and higher values reflect better performance.

### Data Analysis

Principal components analyses (PCA) were used *post hoc* to determine the associations among the dependent measures from all six assessments. PCA is a data mining procedure that finds the best linear fit to the data using a series of perpendicular vectors or principal components (PCs; Clewley et al., 2008). Within each PC vector (i.e., column) the structure of the correlations and non-zero numerical values in each column quantify the relative positive or negative correlations among variables (Clewley et al., 2008). To put it simply, we used PCA as a method of dimensionality reduction that, in this case, examines the contributions of the dependent measures to hand function and the associations among these measures. Due to the differences in units and normal distributions among variables, and for comparison purposes, we calculated the standard score (*z*-score) of each variable and used their standardized normal distribution values for the PCA dataset (Jolliffe, 2005). The PCs are presented in descending order quantifying their contributions to hand function such that the first principal component explained the largest amount of variance. We note that the first three PCs sufficed to capture approximately 85% of the total variance for both datasets; therefore, we limited our analysis to them. Significance was set at *p* < 0.05 and Matlab and SPSS (version 22, IBM, Armonk, NY) was implemented for these analyses.

## Results

The means, standard deviations, and ranges of each dependent measure are presented in Table 1. Clinical outcome measures in all healthy participants were within normal ranges when compared to previously published data (Mathiowetz et al., 1985a,b; Oxford Grice et al., 2003).

**Table 1:** **Outcomes measures from all participants**.

Outcome Measure	Performance	Mean ± SD		Range	
		Healthy	CMC OA	Healthy	CMC OA
Grip (kg)	Higher is better	29.9 ± 13.8	17.1 ± 5.6	5.7–74.5	3.1–31.8
Key Pinch (kg)	Higher is better	7.9 ± 2.6	5.1 ± 1.7	2.7–14.9	2.0–11.0
Precision Pinch (kg)	Higher is better	6 ± 2.4	5.3 ± 1.7	2.3–14.1	2.5–12
Box and Blocks (BBT) (score)	Higher is better	59.2 ± 11.9	55.4 ± 8.8	34–86	29–71
Nine Hole Peg (NHPT) (s)	Lower is better	18.1 ± 5.7	21.5 ± 5.5	9.8–33.7	15.6–48
Strength-Dexterity (SD) (g)	Higher is better	171.4 ± 42.9	170 ± 39.8	83.5–271.4	101.7–245.2

The PCA results from the healthy participants are presented in numerical form below (Table 2). Loading values quantify the strength and direction of the relationships between variables and range between −1 and 1, where 1 is total positive correlation, 0 is no correlation, and −1 is total negative correlation.

**Table 2:** **Association and dissociation of outcome measures in healthy older adults**.

Metric	1st PC	2nd PC	3rd PC
Grip	0.86	−0.61	−0.04
Key Pinch	**1.00**	−0.24	−0.11
Prec. Pinch	0.88	−0.25	−0.54
BBT	0.48	**1.00**	−0.11
*NHPT*	−0.53	−0.99	0.02
*SD*	0.68	−0.05	**1.00**
% Contribution	**47.91%**	**25.03%**	**12.83%**
Cumulative	**47.91%**	**72.94%**	**85.77%**

*Normalized loadings for ease of comparison, Underlining in each column indicates strong (≥0.40) positive and negative correlations, respectively, with the dominant outcome measure, in bold*.

**Figure 1:**
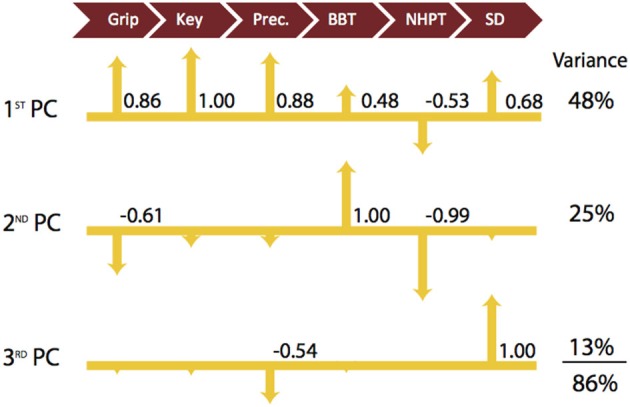
**Visualization of Latent Functional Domains in Healthy Older Adults**. The scaled loadings for the outcome measures of the first three PCs are illustrated above. All loadings are shown, but numerical values are only listed if they are ≥ ±0.40. The signs of the loadings are indicated by the direction of the arrowheads. Note that a higher score is better for all test except for NHPT, where lower is better.

**Figure 2:**
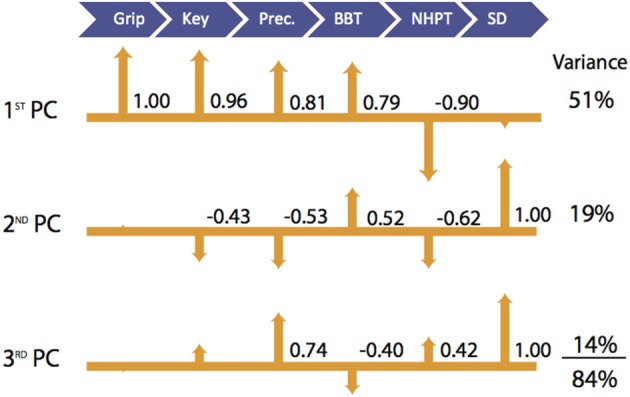
**Visualization of Latent Functional Domains in Participants with CMC OA**. The scaled loadings for the outcome measures of the first three PCs are illustrated above. All loadings are shown, but numerical values are only listed if they are ≥ ±0.40. The signs of the loadings are indicated by the direction of the arrowheads.

The 1st PC explains 48% of the variance and shows that the strength measures are the leading factors distinguishing participants. Key pinch strength representing the highest loading is positively associated with grip strength and precision pinch strength (0.86 and 0.88, respectively). The strength measures were also moderately positively correlated with BBT and SD performance (0.48 and 0.68, respectively) and negatively associated with NHPT (−0.53). The 2nd PC, which explains an additional 25% of the variance, indicates that coordinated upper limb function (BBT) is negatively associated with finger dexterity (NHPT) and grip strength (1.00 vs. −0.99 and −0.61). Furthermore, the 3rd PC explains another 13% of the variance, and indicates that sensorimotor coordination (SD) is the sole contributor and is negatively associated with precision pinch (−0.54). To further explain our results, we provide a visual representation of the respective loadings for each of the first three PCs, Table 2 below in Figure 1. We then repeated our analysis in a group of participants diagnosed with and treated for CMC OA. Those results are presented numerically in Table 3 and visually in Figure 2.

**Table 3:** **Association and dissociation of outcome measures in in older adults with thumb CMC OA**.

Metric	1st PC	2nd PC	3rd PC
Grip	**1.00**	0.04	−0.04
Key Pinch	0.96	−0.43	0.32
Prec. Pinch	0.81	−0.53	0.74
BBT	0.79	0.62	−0.40
*NHPT*	−0.90	−0.52	0.42
*SD*	−0.17	**1.00**	**1.00**
% Contribution	**50.54%**	**19.38%**	**14.08%**
Cumulative	**50.54%**	**69.92%**	**84.01%**

*Normalized loadings for ease of comparison, Underlining in each column indicates strong (≥0.40) positive and negative correlations, respectively, with the dominant outcome measure, in bold*.

In participants with CMC OA, the 1st PC accounted for 51% of the total variance and revealed that outcome measures of hand strength (grip, key pinch, and precision pinch) again demonstrate the highest positive associations (1.00–0.81, respectively). We further report positive and negative associations with BBT (0.79) and NHPT (−0.90). The 2nd PC explained an additional 19% of the variance and indicated that sensorimotor processing (SD test) was the sole contributor and showed moderate associations with measures of finger strength (−0.43 and −0.53) and coordinated upper extremity function (0.62 and −0.52). The SD test again demonstrated the highest loading in the 3rd PC, which explained 14% of the total variance. Additionally, we report a moderate positive association with precision pinch and NHPT (0.74 and 0.42) and a negative association with BBT (−0.40).

## Discussion

Understanding the latent domains of hand function has important implications for both the basic and clinical research communities. The multidimensional ICF model underscores the need to examine outcome measures across the three ICF domains, while at the same time, mapping them to meaningful functional domains. This holds especially true when considering the highly complex nature of the hand and its impact on activity and quality of life. Therefore we applied a dimensionality reduction technique (e.g., PCA) to datasets from six hand-specific outcome measures to determine if and how they mapped into distinct functional domains. We find that the associations and disassociations among the six measures we included reveal three interpretable latent domains of hand function in older adults with and without CMC OA defined as strength, coordinated upper extremity function, and sensorimotor processing. It goes without saying that, although we do not go into detail in this publication, it is important to also consider the inherent psychometric properties (e.g., level of measurement, reliability, validity, etc.) of outcome measures when using them as assessment tools. We note that in this study all outcome measures have been previously shown to be reliable and valid (see Section Material and Methods for more detail).

In healthy older adult participants, 86% of the variance in hand function was explained by the first three PCs with each individually contributing to between 13 and 48% of the total variance. The 4th and higher PCs each contributed to relatively small percentages (4–9%) of total variance and were not considered in our analysis due to the potential for over interpretation. Not surprisingly, the 1st PC indicates that the three hand strength measures tend to be positively associated with each other (Table 2; Figure 1) and that participants tend to vary most in their strength scores (i.e., because most variance is captured by the 1st PC). Thus both hand and finger strength may be most susceptible to age- and health-related declines as they showed the greatest variability among participants. We also find that there are moderate associations between the measures of strength and those of coordinated upper extremity function and sensorimotor coordination. This supports the notion that, while not critical, at least a low-level of strength is required for (and correlated with) successful completion of daily activities and functional tasks (Skelton et al., 1994). There are mixed reports about the contributions of strength to hand function, particularly in older adults. Some have reported improvements in both maximal force production and hand function after exposure to exercise training regimens (Dellhag et al., 1992; Brorsson et al., 2009). In contrast, others report no correlation between the level of force production and the ability to open everyday containers (Rice et al., 1998; Rahman et al., 2002). This agrees with a report that maximal strength is likely not a critical determinant of daily activities because they often require low force magnitudes (Smaby et al., 2004).

In our study, healthy older adults were then best distinguished by tests of coordinated upper extremity function (BBT and NHPT; Figure 1, 2nd PC). The 2nd PC accounted for an additional 25% of the variance and revealed negative associations with measures of strength and little, if any, association with sensorimotor processing. Tests of whole arm function do just that—measure whole arm function. As a result, it is natural to expect that they will not be as informative of hand function *per se* in individuals with, for example, some level of shoulder or elbow dysfunction. This is not a new problem, and has been addressed by many groups (Light et al., 1999, 2002; Duff et al., 2015), which led to the development of specialized devices with the intention of isolating the hand from the arm (Memberg and Crago, 1995). The usefulness of outcome measures featuring such devices is often questioned as they tend to be specialized for certain hand tasks, making their use as a widespread assessment of general hand function ultimately uninformative (Light et al., 1999). Therefore, when evaluating fine motor control, researchers and clinicians often turn to the NHPT, a reliable and validated measure of hand dexterity. Nevertheless, the information obtained from this measure tends to be limited to one’s ability to pick up and place pegs into a board, rather than provide information about sensorimotor coordination or precision strength, and that specificity likely limits its potential for providing basic information on overall hand function (Duff et al., 2015). Moreover, the low and negative correlations among all other outcome measures in the 2nd PC support our prior work where we show that whole-arm function is independent of strength and sensorimotor ability.

The 3rd PC explained another 13% of the variance and also strongly suggested that the SD test captured a different functional domain than either of the other two, likely sensorimotor coordination as our prior work has shown (Valero-Cuevas et al., 2003; Vollmer et al., 2010; Dayanidhi and Valero-Cuevas, 2014). The intricacy of the sensorimotor system dictates that it cannot be quantified with a discreet value or score as with outcome measures geared towards strength or even coordinated upper extremity function. Therefore, one should consider the inclusion of more intricate methods to investigate sensorimotor ability that are decoupled from strength or whole arm function as much as possible in order to not dilute the information gained (Valero-Cuevas et al., 2003). As such, SD test offers a means to quantify the dynamic interaction between fingertip force magnitudes and directions during a dynamic sub-maximal pinch task, which we have shown is informative of sensorimotor ability (Valero-Cuevas et al., 2003; Talati et al., 2005; Venkadesan et al., 2007; Vollmer et al., 2010; Holmström et al., 2011; Dayanidhi et al., 2013; Dayanidhi and Valero-Cuevas, 2014; Lawrence et al., 2014; Lightdale-Miric et al., 2015; Duff et al., 2015).

We find evidence in our results that support the fact that sensorimotor processing is distinct from strength or coordinated upper limb function. For example, notice that the SD test is independent of grip and key pinch strength (Figure 1, 3rd PC), and moderately negatively correlated with precision pinch strength (−0.54) in the same finger posture (i.e., tip-to-tip pinch). This complements our prior work that shows that declines in strength and dexterous manipulation are disassociated in older adults (Dayanidhi and Valero-Cuevas, 2014). Recall that the SD test, by using compliant slender springs, requires only very low forces in the order of 3N. Thus although the “greatest mean compression force” is the measured variable, in reality the level of force is indicative of the maximal instability that can be controlled at low force levels. Secondly, this interpretation of a distinct functional domain of sensorimotor processing is consistent with functional magnetic resonance imaging (fMRI) studies showing that: (1) force production and stabilization, two main features of dexterous manipulation, are represented by two distinct areas within the grasping network (Holmström et al., 2011); and (2) the areas of activation in the sensorimotor cortices are dependent on task dexterity requirements (Mosier et al., 2011). Finally, in the 3rd PC, the SD test showed no association with either the BBT or the NHPT (Figure 1). This combined with the lack of association in the 2nd PC that we discussed previously supports the notion that sensorimotor processing represents a domain of hand function not strongly correlated with coordinated upper limb function. These results mirror our prior work pertaining to the development of dexterity in children where sensorimotor processing was found to be a functional dimension distinctly different from strength and whole arm coordination (Vollmer et al., 2010).

Our study also allowed us to investigate the contributions of each domain of hand function in a group of older adults affected by thumb CMC OA. The first three PCs suffice to explain 84% of the total variance in hand function; therefore, we limit our interpretations to them. Interestingly, the associations among outcome measures found in healthy adults were altered in the presence of thumb CMC OA. The latent functional domains of strength and coordinated upper extremity function seem to merge and show no association with sensorimotor processing in the 1st PC (Figure 2), which explained 51% of the total variance. This suggests that in the presence of the physiological effects of thumb CMC OA, upper extremity coordination is no longer its own independent domain and becomes strongly associated with strength, while dynamic sensorimotor ability remains an independent domain. Sensorimotor processing is the leading contributor in the 2nd PC (Figure 2) and showed moderate associations with outcome measures associated with finger strength (precision and key pinch) and coordinated upper extremity function (NHPT and BBT) that were not present in the healthy participants. This may suggest that the reductions of both strength and coordinated upper extremity function often associated with thumb CMC OA (Bagis et al., 2003; Dominick et al., 2005; Kjeken et al., 2005) place greater emphasis on sensorimotor processing as a compensatory strategy for successful hand function. We further report a positive association of the SD test, which dominated the 3rd PC, with precision pinch strength (0.74) in participants with thumb CMC OA (Figure 2, 3rd PC), unlike in healthy participants where we report a moderately negative association (−0.54; Figure 1, 3rd PC). This suggests that the pain and anatomical deformities associated with thumb CMC OA may also alter the association between the strength and sensorimotor processing latent domains.

It is important to note that the participants with thumb CMC OA were all female, while the healthy older adult group was both male and female to accurately represent the older adult population. We chose to only test women in the clinical group because thumb CMC OA is disproportionately more prevalent in women, starting at the fifth decade of life (Armstrong et al., 1994; Comtet et al., 2001; Haara et al., 2004); thus finding suitable male candidates would have been difficult, but also would have potentially introduced a sex effect in the SD test that we have reported in the past Lawrence et al. (2014). For these reasons, we also ran our PCA separately for female and male healthy participants to compare against the all female thumb CMC OA group. While we do not show those results for succinctness, we found that the PCs found in the combined group of healthy participants remained unchanged when analyzing the data from only males or females. This gives us confidence that the differences we report between groups can, in fact, be attributed to the presence of thumb CMC OA.

How clinically informative of hand function are the three latent domains of hand function that we found? We argue that they are very informative because they are inherently compatible with ICF classifications of body structure and function, activity and participation, and inform those classifications with specific experimental data. That is, strength and sensorimotor processing fit within the structure and function category; and coordinated upper extremity function fits within activity (reach to grasp) and participation (necessary for work, play and ADLs); however it is not as clear in case of the patients with OA where the domains are muddled. We note that the ICF itself recognizes that these classifications are not exclusive because strength is often needed for work and sensorimotor processing is needed to perform in-hand manipulation once objects are picked up, etc. Nevertheless, in our minds, our results do provide specificity to the ICF criteria in the context of hand function by providing a link to real-world outcome measures.

But most importantly, these three functional domains emerged naturally from the data. As such, our methodology provides a window into latent contributors to hand function and means to quantify them. This ability to naturally identify and quantify functional domains allows us to probe the underlying physiological mechanisms that enable, impair, or restore general manipulation ability in everyday life, particularly with respect to healthy aging and aging with a disability. By corroborating the existence of these three functional domains in older adults that we had seen in children, these results suggest that they are present throughout the lifespan—and are therefore an inherent property of human hands. The presence of these three latent domains in both development and aging motivates their study throughout the lifespan.

Understanding effects of aging on quality of life is now emerging as an important public health issue (Verbrugge et al., 1991; Kemp and Mosqueda, 2004; Covinsky, 2006; Song et al., 2006; Winstein et al., 2012). It becomes even more so when we consider the added orthopedic and/or neurological effects when aging with—or into—a disability. In fact, we have a prior publication showing that both CMC OA and Parkinson’s pathology exacerbates the aging effect (Lawrence et al., 2014). As an extension, in this paper we focused on understanding the latent domains of functions in the context of healthy aging and aging with a disability. For example, our results suggest an underappreciated and understudied link between what is at its core a disease of articular cartilage, and sensorimotor integration capabilities for dexterous manipulation. This ability to quantify and describe functional domains should play a central role when quantifying age-related losses in hand function in general; and in particulate help us understand and optimize treatments for thumb CMC OA and other orthopedic and neurological conditions in our aging populations.

## Conflict of Interest Statement

FVC holds US Patent No. 6,537,075 on some of the technology used, but has no active or pending licensing agreements with any commercial entity. None of the other authors have any financial or personal relationships with other people or organizations that could inappropriately influence this work.

## References

[B1] Armstrong A. L. Hunter J. B. Davis T. R. (1994). The prevalence of degenerative arthritis of the base of the thumb in post-menopausal women. J. Hand Surg. Br. 19, 340–341. 10.1016/0266-7681(94)90085-x 8077824

[B2] Bagis S. Sahin G. Yapici Y. Cimen O. B. Erdogan C. (2003). The effect of hand osteoarthritis on grip and pinch strength and hand function in postmenopausal women. Clin. Rheumatol. 22, 420–424. 10.1007/s10067-003-0792-4 14677019

[B3] Boyle A. M. Santelli J. C. (1986). Assessing psychomotor skills: the role of the Crawford small parts dexterity test as a screening instrument. J. Dent. Educ. 50, 176–179. 3456367

[B4] Brorsson S. Hilliges M. Sollerman C. Nilsdotter A. (2009). A six-week hand exercise programme improves strength and hand function in patients with rheumatoid arthritis. J. Rehabil. Med. 41, 338–342. 10.2340/16501977-0334 19363566

[B5] Clewley R. H. Guckenheimer J. M. Valero-Cuevas F. J. (2008). Estimating effective degrees of freedom in motor systems. IEEE Trans. Biomed. Eng. 55, 430–442. 10.1109/TBME.2007.903712 18269978

[B6] Comtet J. J. Gazarian A. Fockens W. (2001). [Definition and classification of basal joint osteoarthritis. A critical analysis and proposals. Treatment options]. Chir. Main 20, 5–10. 10.1016/S1297-3203(01)00009-9 11291319

[B7] Covinsky K. (2006). Aging, arthritis and disability. Arthritis Rheum. 55, 175–176. 10.1002/art.21861 16583390

[B8] Cromwell F. S. (1976). Occupational Therapist’s Manual for Basic Skill Assessment; Primary Prevocational Evaluation. Fair Oaks, CA: Fair Oaks Printing.

[B10] Dayanidhi S. Hedberg A. Valero-Cuevas F. J. Forssberg H. (2013). Developmental improvements in dynamic control of fingertip forces last throughout childhood and into adolescence. J. Neurophysiol. 110, 1583–1592. 10.1152/jn.00320.2013 23864371 PMC4042419

[B9] Dayanidhi S. Valero-Cuevas F. J. (2014). Dexterous manipulation is poorer at older ages and is dissociated from decline of hand strength. J. Gerontol. A Biol. Sci. Med. Sci. 69, 1139–1145. 10.1093/gerona/glu025 24610868 PMC4202259

[B11] Dellhag B. Wollersjö I. Bjelle A. (1992). Effect of active hand exercise and wax bath treatment in rheumatoid arthritis patients. Arthritis Care Res. 5, 87–92. 10.1002/art.1790050207 1390969

[B12] Dellon A. L. Mackinnon S. E. Crosby P. M. (1987). Reliability of two-point discrimination measurements. J. Hand Surg. Am. 12, 693–696. 10.1016/s0363-5023(87)80049-7 3655225

[B13] Desrosiers J. Hébert R. Bravo G. Dutil E. (1995). Upper extremity performance test for the elderly (TEMPA): normative data and correlates with sensorimotor parameters. Test d’Evaluation des membres superieurs de personnes agees. Arch. Phys. Med. Rehabil. 76, 1125–1129. 10.1016/s0003-9993(95)80120-0 8540788

[B14] Dominick K. L. Jordan J. M. Renner J. B. Kraus V. B. (2005). Relationship of radiographic and clinical variables to pinch and grip strength among individuals with osteoarthritis. Arthritis Rheum. 52, 1424–1430. 10.1002/art.21035 15880347

[B15] Duff S. V. Aaron D. H. Gogola G. R. Valero-Cuevas F. J. (2015). Innovative evaluation of dexterity in pediatrics. J. Hand Ther. 28, 144–150. 10.1016/j.jht.2015.01.004 25835255 PMC4424153

[B16] Egan M. Davis C. G. Dubouloz C. J. Kessler D. Kubina L. A. (2014). Participation and well-being poststroke: evidence of reciprocal effects. Arch. Phys. Med. Rehabil. 95, 262–268. 10.1016/j.apmr.2013.08.013 24001446

[B17] Haara M. M. Heliövaara M. Kröger H. Arokoski J. P. Manninen P. Kärkkäinen A.. (2004). Osteoarthritis in the carpometacarpal joint of the thumb. Prevalence and associations with disability and mortality. J. Bone Joint Surg. Am. 86-A, 1452–1457. 15252092 10.2106/00004623-200407000-00013

[B18] Hart T. Bagiella E. (2012). Design and implementation of clinical trials in rehabilitation research. Arch. Phys. Med. Rehabil. 93, S117–S126. 10.1016/j.apmr.2011.11.039 22840878

[B19] Hellström K. Lindmark B. Wahlberg B. Fugl-Meyer A. R. (2003). Self-efficacy in relation to impairments and activities of daily living disability in elderly patients with stroke: a prospective investigation. J. Rehabil. Med. 35, 202–207. 10.1080/16501970310000836 14582550

[B20] Holmström L. de Manzano O. Vollmer B. Forsman L. Valero-Cuevas F. Ullén F.. (2011). Dissociation of brain areas associated with force production and stabilization during manipulation of unstable objects. Exp. Brain Res. 215, 359–367. 10.1007/s00221-011-2903-9 22038714 PMC3950331

[B21] Hume M. C. Gellman H. McKellop H. Brumfield R. H. Jr. (1990). Functional range of motion of the joints of the hand. J. Hand Surg. Am. 15, 240–243. 10.1016/0363-5023(90)90102-w 2324451

[B22] Jebsen R. H. Taylor N. Trieschmann R. B. Trotter M. J. Howard L. A. (1969). An objective and standardized test of hand function. Arch. Phys. Med. Rehabil. 50, 311–319. 5788487

[B23] Jolliffe I. (2005). “Principal component analysis,” in Encyclopedia of Statistics in Behavioral Science, eds Everitt B. Howell D. (New York, NY: John Wiley and Sons, Ltd.).

[B24] Kemp B. J. Mosqueda L. (2004). Aging with a Disability: What the Clinician Needs to Know. Baltimore, MD: JHU Press.

[B25] Kjeken I. Dagfinrud H. Slatkowsky-Christensen B. Mowinckel P. Uhlig T. Kvien T. K.. (2005). Activity limitations and participation restrictions in women with hand osteoarthritis: patients’ descriptions and associations between dimensions of functioning. Ann. Rheum. Dis. 64, 1633–1638. 10.1136/ard.2004.034900 15829571 PMC1755278

[B26] Lawrence E. L. Fassola I. Werner I. Leclercq C. Valero-Cuevas F. J. (2014). Quantification of dexterity as the dynamical regulation of instabilities: comparisons across gender, age and disease. Front. Neurol. 5:53. 10.3389/fneur.2014.00053 24782824 PMC3995042

[B27] Light C. M. Chappell P. H. Kyberd P. J. (2002). Establishing a standardized clinical assessment tool of pathologic and prosthetic hand function: normative data, reliability and validity. Arch. Phys. Med. Rehabil. 83, 776–783. 10.1053/apmr.2002.32737 12048655

[B28] Light C. M. Chappell P. H. Kyberd P. J. Ellis B. S. (1999). A critical review of functionality assessment in natural and prosthetic hands. Br. J. Occup. Ther. 62, 7–12. 10.1177/030802269906200103

[B29] Lightdale-Miric N. Mueske N. M. Dayanidhi S. Loiselle J. Berggren J. Lawrence E. L.. (2015). Quantitative assessment of dynamic control of fingertip forces after pollicization. Gait Posture 41, 1–6. 10.1016/j.gaitpost.2014.08.012 25262333 PMC4267977

[B30] Marx R. G. Bombardier C. Wright J. G. (1999). What do we know about the reliability and validity of physical examination tests used to examine the upper extremity? J. Hand Surg. Am. 24, 185–193. 10.1053/jhsu.1999.jhsu24a0185 10048536

[B31] Mathiowetz V. Kashman N. Volland G. Weber K. Dowe M. Rogers S. (1985a). Grip and pinch strength: normative data for adults. Arch. Phys. Med. Rehabil. 66, 69–74. 3970660

[B32] Mathiowetz V. Volland G. Kashman N. Weber K. (1985b). Adult norms for the box and block test of manual dexterity. Am. J. Occup. Ther. 39, 386–391. 10.5014/ajot.39.6.386 3160243

[B33] McDonough C. M. Jette A. M. Ni P. Bogusz K. Marfeo E. E. Brandt D. E.. (2013). Development of a self-report physical function instrument for disability assessment: item pool construction and factor analysis. Arch. Phys. Med. Rehabil. 94, 1653–1660. 10.1016/j.apmr.2013.03.011 23542402 PMC4046327

[B34] Memberg W. D. Crago P. E. (1995). A grasp force and position sensor for the quantitative evaluation of neuroprosthetic hand grasp systems. IEEE Trans. Rehabil. Eng. 3, 175–181. 10.1109/86.392370

[B35] Miller K. J. Phillips B. A. Martin C. L. Wheat H. E. Goodwin A. W. Galea M. P. (2009). The AsTex: clinimetric properties of a new tool for evaluating hand sensation following stroke. Clin. Rehabil. 23, 1104–1115. 10.1177/0269215509342331 19897517

[B36] Milot M. H. Spencer S. J. Chan V. Allington J. P. Klein J. Chou C.. (2013). A crossover pilot study evaluating the functional outcomes of two different types of robotic movement training in chronic stroke survivors using the arm exoskeleton BONES. J. Neuroeng. Rehabil. 10:112. 10.1186/1743-0003-10-112 24354476 PMC3878268

[B37] Mosier K. Lau C. Wang Y. Venkadesan M. Valero-Cuevas F. (2011). Controlling instabilities in manipulation requires specific cortical-striatal-cerebellar networks. J. Neurophysiol. 105, 1295–1305. 10.1152/jn.00757.2010 21228301 PMC3074419

[B38] Oxford Grice K. Vogel K. A. Le V. Mitchell A. Muniz S. Vollmer M. A. (2003). Adult norms for a commercially available Nine Hole Peg Test for finger dexterity. Am. J. Occup. Ther. 57, 570–573. 10.5014/ajot.57.5.570 14527120

[B39] Patterson K. K. Gage W. H. Brooks D. Black S. E. Mcilroy W. E. (2010). Changes in gait symmetry and velocity after stroke: a cross-sectional study from weeks to years after stroke. Neurorehabil. Neural Repair 24, 783–790. 10.1177/1545968310372091 20841442

[B40] Rahman N. Thomas J. J. Rice M. S. (2002). The relationship between hand strength and the forces used to access containers by well elderly persons. Am. J. Occup. Ther. 56, 78–85. 10.5014/ajot.56.1.78 11833404

[B41] Rice M. S. Leonard C. Carter M. (1998). Grip strengths and required forces in accessing everyday containers in a normal population. Am. J. Occup. Ther. 52, 621–626. 10.5014/ajot.52.8.621 9739394

[B42] Skelton D. A. Greig C. A. Davies J. M. Young A. (1994). Strength, power and related functional ability of healthy people aged 65–89 years. Age Ageing 23, 371–377. 10.1093/ageing/23.5.371 7825481

[B43] Smaby N. Johanson M. E. Baker B. Kenney D. E. Murray W. M. Hentz V. R. (2004). Identification of key pinch forces required to complete functional tasks. J. Rehabil. Res. Dev. 41, 215–224. 10.1682/jrrd.2004.02.0215 15558375

[B44] Smith D. A. (1961). The Box and Block Test: Normative Data for 7, 8, 9 Year Old Children. Los Angeles, CA: University of Southern California.

[B45] Song J. Chang R. W. Dunlop D. D. (2006). Population impact of arthritis on disability in older adults. Arthritis Rheum. 55, 248–255. 10.1002/art.21842 16583415 PMC2757646

[B46] Talati A. Valero-Cuevas F. Hirsch J. (2005). Visual and tactile guidance of dexterous manipulation tasks: an fMRI study. Percept. Mot. Skills 101, 317–334. 10.2466/pms.101.5.317-334 16353365

[B47] Trombly C. A. (1983). Occupational Therapy for Physical Dysfunction. Philadelphia, PA: Lippincott Williams, and Wilkins.

[B48] Valero-Cuevas F. Smaby N. Venkadesan M. Peterson M. Wright T. (2003). The strength-dexterity test as a measure of dynamic pinch performance. J. Biomech. 36, 265–270. 10.1016/s0021-9290(02)00340-8 12547365

[B49] van Lankveld W. van’t Pad Bosch P. Bakker J. Terwindt S. Franssen M. van Riel P. (1996). Sequential occupational dexterity assessment (SODA): a new test to measure hand disability. J. Hand Ther. 9, 27–32. 10.1016/s0894-1130(96)80008-1 8664936

[B50] Venkadesan M. Guckenheimer J. Valero-Cuevas F. (2007). Manipulating the edge of instability. J. Biomech. 40, 1653–1661. 10.1016/j.jbiomech.2007.01.022 17400231 PMC2666355

[B51] Verbrugge L. M. Lepkowski J. M. Konkol L. L. (1991). Levels of disability among U.S. adults with arthritis. J. Gerontol. 46, S71–S83. 10.1093/geronj/46.2.s71 1997585

[B52] Vollmer B. Holmström L. Forsman L. Krumlinde-Sundholm L. Valero-Cuevas F. Forssberg H.. (2010). Evidence of validity in a new method for measurement of dexterity in children and adolescents. Dev. Med. Child Neurol. 52, 948–954. 10.1111/j.1469-8749.2010.03697.x 20497459 PMC3080099

[B53] Walker P. S. Davidson W. Erkman M. J. (1978). An apparatus to assess function of the hand. J. Hand Surg. Am. 3, 189–193. 10.1016/s0363-5023(78)80072-0 632551

[B54] Winstein C. J. Requejo P. S. Zelinski E. M. Mulroy S. J. Crimmins E. M. (2012). A transformative subfield in rehabilitation science at the nexus of new technologies, aging and disability. Front. Psychol. 3:340. 10.3389/fpsyg.2012.00340 23049517 PMC3448347

[B55] World Health Organization (2001). International Classification of Functioning, Disability and Health. New York, NY: World Health Organization.

